# The EvIDHence study: real-world treatment patterns and safety outcomes in mutant and wild-type *IDH1* cholangiocarcinoma before availability of mIDH1 inhibitors

**DOI:** 10.1016/j.esmogo.2026.100367

**Published:** 2026-07-23

**Authors:** V. Sahai, J.W. Valle, N. Fares, M. Javle, I. Ghanem, P. Cerdà, I.G. Rapposelli, I.G. Jimenez, M.A. Gonzalez-Carmona, A. Hollebecque, R. Pazo Cid, D.H. Palmer, R. Vera, A. Casadei-Gardini, D. Melisi, J.-F. Blanc, L. Perkhofer, A. Lamarca, H. Gharbi, V. Manuel-Minguez, R. Robert, L. Rimassa

**Affiliations:** 1 Division of Hematology and Oncology, Department of Internal Medicine, University of Michigan, Ann Arbor; 2 Cholangiocarcinoma Foundation, Salt Lake City, USA; 3 Department of Medical Oncology, Division of Cancer Sciences, University of Manchester, Manchester, UK; 4 Digestive Oncology Unit, Rangueil University Hospital, CHU Toulouse, Toulouse, France; 5 Department of Gastrointestinal Medical Oncology, Division of Cancer Medicine, MD Anderson Cancer Center, Houston, USA; 6 Department of Medical Oncology, Hospital Universitario La Paz, Madrid, Spain; 7 Medical Oncology Department, Instituto de Investigación Sanitaria del Hospital Universitario La Paz (IdiPAZ), Madrid, Spain; 8 Medical Oncology Department, Hospital de la Santa Creu i Sant Pau, Barcelona, Spain; 9 Department of Medical Oncology, IRCCS Istituto Romagnolo per lo Studio dei Tumori (IRST) “Dino Amadori”, Meldola, Italy; 10 Medical Oncology Department, Virgen del Rocío University Hospital, IBIS, Seville, Spain; 11 Department of Medicine I, University Hospital of Bonn, Bonn, Germany; 12 Early Drug Development Department, Gustave Roussy, Villejuif, France; 13 Medical Oncology Department, Hospital Universitario Miguel Servet, Saragossa, Spain; 14 Department of Molecular and Clinical Cancer Medicine, Cancer Research UK Liverpool Experimental Cancer Medicine Centre, University of Liverpool and The Clatterbridge Cancer Centre, Liverpool, UK; 15 Medical Oncology Department, University Hospital of Navarra, Instituto de Investigación Sanitaria de Navarra, IdISNA, Pamplona, Navarra, Spain; 16 Department of Oncology, Vita-Salute San Raffaele University, IRCCS San Raffaele Scientific Institute Hospital, Milan, Italy; 17 Investigational Cancer Therapeutics Clinical Unit, Università degli Studi di Verona and Azienda Ospedaliera Universitaria Integrata, Verona, Italy; 18 Digestive Oncology Department, University Hospital of Bordeaux, Bordeaux, France; 19 Department of Internal Medicine 1, University Hospital Ulm, Ulm, Germany; 20 Department of Internal Medicine, Institute of Molecular Oncology and Stem Cell Biology, Ulm University Hospital, Ulm, Germany; 21 Department of Medical Oncology, Hospital Universitario Fundación Jiménez Díaz, Madrid, Spain; 22 Medical Affairs, Servier, Suresnes, France; 23 Department of Biomedical Sciences, Humanitas University, Pieve Emanuele, Milan, Italy; 24 Department of HepatoPancreatoBiliary Oncology, Humanitas Cancer Center, IRCCS Humanitas Research Hospital, Rozzano, Milan, Italy

**Keywords:** biomarker testing, cholangiocarcinoma, isocitrate dehydrogenase-1, safety, survival, treatment patterns

## Abstract

**Background:**

Treatment options for cholangiocarcinoma (CCA) are limited, with poor overall survival (OS). The impact of molecular alterations on clinical outcomes in real-world practice is poorly understood.

**Patients and Methods:**

EvIDHence was a retrospective, descriptive, multicenter, international, chart review study investigating the natural disease course of patients with advanced/metastatic CCA who underwent mutant isocitrate dehydrogenase-1 (m*IDH1*) testing and initiated second-line treatment on or before 1 July 2019 until 31 December 2021, before commercial availability of mIDH1-targeted therapy. Outcomes included patient, disease, and treatment characteristics, biomarker testing and survival.

**Results:**

Sixty-three patients with CCA (31 m*IDH1*; 32 *IDH1* wild-type) were included. There were no differences in patient and disease characteristics between m*IDH1* and *IDH1* wild-type groups. Biomarker testing was carried out in the context of research/clinical trials in 61.9% of patients. The most frequent first- and second-line systemic regimens were gemcitabine plus cisplatin and FOLFOX, respectively. Median OS from first-line treatment initiation was 20.8 [95% confidence interval (CI) 15.1-29.3] and 18.6 (95% CI 13.0-20.5) months for m*IDH1* and *IDH1* wild-type groups, respectively. Median OS from second-line treatment initiation was 9.7 (95% CI 5.5-11.2) and 8.5 (95% CI 6.5-11.3) months for patients with m*IDH1* and *IDH1* wild-type CCA, respectively.

**Conclusion:**

These real-world data highlight the poor prognosis of patients with CCA, regardless of *IDH1* mutational status. Limitations included the small sample size and biased selection of patients still fit for second-line therapy.

## Highlights

• EvIDHence investigated real-world treatment patterns and outcomes of patients with CCA.
• These outcomes were assessed in patients with mutant and wild-type *IDH1* CCA before the availability of mIDH1 inhibitors.
• Disease and patient characteristics were similar between patients with mutant and wild-type *IDH1* CCA.
• The most frequent systemic regimen used was gemcitabine + cisplatin as first-line and FOLFOX as second-line therapy.
• Median OS from therapy initiation was similar between mutant *IDH1* (20.8 months) and *IDH1* wild-type (18.6 months) groups.

## Introduction

Cholangiocarcinoma (CCA) is the most common biliary tract malignancy and the second most common hepatic malignancy after hepatocellular carcinoma.^1^ Treatment options for CCA are limited, and those that are available are associated with poor survival outcomes. Curative surgery and liver transplantation are feasible for a small proportion of patients; however, the rate of recurrence has been reported to be as high as 65%.^2^ The five-year survival rate is ∼25% for patients with operable disease and <5% for patients with advanced CCA.^3^

At the time of the study reported in this paper, the first-line (1L) treatment choice for patients with advanced unresectable or metastatic CCA was gemcitabine plus cisplatin.^4^ In the 2025 update to the European Society of Medical Oncology (ESMO) guidelines, recommended 1L treatment is gemcitabine plus cisplatin in combination with an immune checkpoint inhibitor (durvalumab or pembrolizumab).^5^ FOLFOX (folinic acid, 5-fluorouracil, oxaliplatin) is the recommended second-line (2L) treatment for patients with advanced or metastatic CCA with no targetable molecular alterations.^5^^,^^6^ Recent advances in drug development and approvals have led to changes in treatment paradigms, with 2L treatment options incorporating targeted therapies for CCA.^6^, ^7^, ^8^, ^9^, ^10^, ^11^, ^12^, ^13^

Mutations in isocitrate dehydrogenase-1 (m*IDH1*) occur in ∼15% of patients with intrahepatic CCA (iCCA), making these patients potential candidates for targeted treatment with mIDH1 inhibitors.^14^ In the phase 3 ClarIDHy trial, ivosidenib, a small-molecule targeted inhibitor of mIDH1, was well tolerated and significantly improved progression-free survival (PFS) compared with placebo in patients with m*IDH1* CCA who had progressed on one to two prior therapies.^8^^,^^9^ Median overall survival (OS) was also significantly improved with ivosidenib compared with placebo when adjusted for patients initially allocated to placebo who later crossed over to ivosidenib following disease progression [hazard ratio (HR) 0.49, 95% confidence interval (CI) 0.34-0.70; *P* < 0.001].^9^ Based on the results from ClarIDHy, ivosidenib was approved in August 2021 in the USA and May 2023 in Europe as monotherapy for adult patients with locally advanced or metastatic CCA with m*IDH1 R132* who were previously treated with ≥1 line of systemic therapy.^15^

These recent approvals have broadened the treatment landscape by incorporating the use of targeted therapy for patients with advanced and metastatic disease harboring an actionable molecular alteration in *IDH1*.^6^, ^7^, ^8^, ^9^, ^10^, ^11^^,^^13^^,^^16^^,^^17^ Despite such advances, further investigation is needed to improve understanding of the impact of major oncogenic mutations and other molecular alterations on prognosis, response to chemotherapy, and optimization of CCA treatment. Thus, it is essential to gather more clinical outcomes and treatment sequence information for populations with both m*IDH1* and *IDH1* wild-type CCA based on real-world data as documented in medical charts.

Therefore, the EvIDHence study aims to fill the gap in knowledge in the real world by describing patient characteristics, biomarker testing techniques, prognostic impact of m*IDH1*, and treatment sequences used in patients with m*IDH1* and *IDH1* wild-type CCA treated with standard-of-care therapies before commercial availability of ivosidenib.

## Patients and methods

### Study design

**Figure 1:**
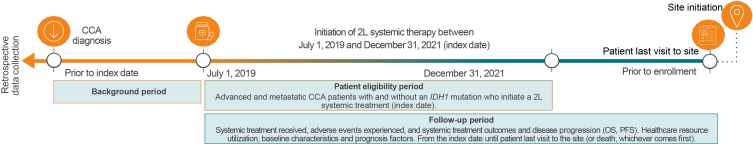
**Study design.** In the USA, the eligibility period was extended beyond 1 July 2019 to include all patients with an index date on or before 31 December 2021. 2L, second-line; CCA, cholangiocarcinoma; *IDH1*, isocitrate dehydrogenase-1; OS, overall survival; PFS, progression-free survival.

EvIDHence was a descriptive, multicenter, international, longitudinal, retrospective, non-interventional study across five European countries (France, Germany, Italy, Spain, and the UK) and the USA (Figure 1). Study investigators extracted retrospective data from the medical records of living or deceased adult patients with advanced CCA, with and without m*IDH1*, who initiated a 2L systemic therapy during the study eligibility period.

### Patients

Adult patients with advanced or metastatic CCA, irrespective of subtype [iCCA, perihilar (pCCA), extrahepatic distal (dCCA), or unknown subtype], who initiated 2L treatment after progression on 1L treatment, between 1 July 2019 and 31 December 2021 inclusive for France, Germany, Italy, Spain, and the UK, and before 31 December 2021 for the USA, were included in this study. Progressive disease on 1L treatment was defined as radiological progression judged by a treating physician as no longer benefiting from the current 1L systemic treatment. Moreover, patients needed a confirmed positive or negative test for m*IDH1* status and availability of 1L and 2L survival data for advanced/metastatic disease in their medical records. Once all eligible patients with m*IDH1* CCA had been identified from each site, an equal number of patients with *IDH1* wild-type CCA were selected from the same center using random letter assignment from a list of all eligible patients with *IDH1* wild-type CCA. Patients who were deceased at the time of enrolment were also eligible for study inclusion.

Patients were excluded if they met any of the following criteria: received any investigational mIDH1 inhibitor at any stage of CCA treatment (including investigational treatments or those marketed in the USA); were not tested for an *IDH1* mutation; lacked documented systemic therapy outcomes or prior treatments in their medical records; or were receiving best supportive care during 2L therapy.

### Data sources

Data were extracted retrospectively from institutional medical records of eligible patients from each participating center. The participating investigators were responsible for the collection and accuracy of data documented within an online electronic case report form for each enrolled patient.

### Assessments

Data were collected throughout the patient eligibility period and follow-up period [from the index date (defined as the date when the patient initiated a 2L systemic therapy) until the last patient visit or death; Figure 1]. Patient characteristics including age, gender, smoking habits, and alcohol consumption were collected. Data on the treatments received, including surgery (type and setting), radiotherapy (type and setting), and systemic therapy (agents/regimens, number of cycles, dose adjustments, treatment duration, treatment interruptions, and reasons for interruptions), were also collected. OS (time from treatment initiation to death) and PFS (time from treatment initiation to next therapy, disease progression, or death, whichever came first) were measured from the start of 1L and 2L. OS and PFS were also calculated in patients receiving a gemcitabine-platinum regimen at 1L and in patients receiving FOLFOX regimen at 2L. Disease characteristics, including CCA subtype, were collected. *IDH* mutation testing characteristics, including methodology used to detect molecular alterations, time to results (turnaround), and co-molecular alterations, were recorded. Data on medical resource utilization, comprising routine site visits, inpatient hospitalizations by unit/department type, outpatient hospitalizations by unit/department type, office visits categorized by physician/specialist type, and imaging examinations, were also collected.

### Study objectives

The study objectives were to identify treatment sequences and survival outcomes in populations with m*IDH1* and *IDH1* wild-type CCA and to describe the patient and tumor characteristics, including molecular alterations and testing. Exploratory objectives included medical resource utilization in populations with m*IDH1* and *IDH1* wild-type CCA in the 2L setting.

Due to the retrospective nature of this study, the reported adverse events were adverse drug reactions only, consistent with the label of the administered regimen.

### Statistical analysis

Given the descriptive nature of this study, sample size calculation was not carried out. The anticipated total sample size was 200 patients, 100 with m*IDH1* and 100 with *IDH1* wild-type CCA. However, the study size was lower than anticipated due to the limited number of patients with m*IDH1* CCA who had undergone biomarker testing and had not received an mIDH1 inhibitor (as ivosidenib was available through a compassionate access program during the enrolment period). Standard descriptive statistics were provided for patient characteristics, clinical features, and disease characteristics. CCA treatment patterns were also analyzed using descriptive statistics at each stage of treatment, including surgery, radiotherapy, and each line of systemic therapy. The following variables were analyzed: regimens of systemic therapy (number and proportion of patients prescribed each regimen), treatment duration (in days), the number of cycles, and dose adjustments (if any), as well as treatment interruptions and the reasons for treatment interruptions.

To assess treatment outcomes in the real-world clinical practice, OS and PFS were analyzed in the total population and in each subgroup (patients with m*IDH1* and *IDH1* wild-type CCA) at 1L and 2L. PFS and OS were analyzed from the start of 1L and 2L using the Kaplan–Meier method. An exploratory analysis assessing the impact of m*IDH1* and *IDH1* wild-type on study outcomes (OS and PFS) was carried out using univariate and multivariate Cox regression models. Variables in the multivariate analysis were FOLFOX in 2L, gemcitabine-platinum regimen in 1L, surgery, age ≤60 years at advanced diagnosis, Eastern Cooperative Oncology Group performance status (ECOG PS), tumor localization, carbohydrate antigen 19 levels, CCA stage, radiation, and gender. Comparisons between subgroups of interest were conducted to determine the impact of patient mutation status (m*IDH1* versus *IDH1* wild-type) on survival outcomes at diagnosis, 1L, and 2L.

Continuous variables were described by sample size (*n*), measures of central tendency (e.g. mean, median), and measures of variation (e.g. standard deviation [SD], minimum, maximum, and quartiles). Categorical variables were described by frequency tables containing the sample size and proportions (%) falling into each category. All analyses were conducted using SAS® software version 9.4 or later.

## Results

### Patient characteristics

Seventy-four patients were screened for eligibility for this study (Supplementary Figure S1, available at https://doi.org/10.1016/j.esmogo.2026.100367), and all patients screened had a confirmed positive or negative test for m*IDH1* status as per the inclusion criterion. Between September 2022 and September 2023, 63 patients from 28 centers were subsequently included in the study: 23 patients from the USA, 13 from Spain, 10 from Italy, 8 from the UK, 7 from France, and 2 from Germany. At inclusion, 56 (88.9%) patients were deceased, with 45 deaths (80.4%) explicitly related to CCA. Of the patients included in this study, 31 had m*IDH1* CCA and 32 had *IDH1* wild-type CCA. Evaluable imaging was available for 23 patients with m*IDH1* CCA and 16 patients with *IDH1* wild-type CCA in 1L and 10 patients with m*IDH1* CCA and 13 patients with *IDH1* wild-type CCA in 2L.

Patient and disease characteristics are summarized in Table 1. The most frequent CCA subtype was intrahepatic in both the *IDH1* wild-type (23/32; 71.9%) and m*IDH1* subgroups (26/31; 83.9%; Table 1). In the *IDH1* wild-type and m*IDH1* subgroups, 18 (56.3%) and 16 (51.6%) patients were female, respectively. The mean (SD) age at initial diagnosis was 59.3 (10.6) years in the *IDH1* wild-type subgroup, and 58.5 (12.0) years in the m*IDH1* subgroup. At 1L, 54 (98.2%) patients had an ECOG PS of 0-1, whereas 44 (93.6%) patients had an ECOG PS of 0-1 at 2L. Among patients with *IDH1* wild-type and m*IDH1* CCA, 18 (56.3%) and 13 (41.9%), respectively, suffered from at least one comorbidity, primarily metabolic conditions. Cirrhosis was reported in three (9.4%) patients with *IDH1* wild-type CCA and one (3.2%) patient with m*IDH1* CCA. Steatotic liver disease occurred in one (3.1%) patient with *IDH1* wild-type CCA and two (6.5%) patients with m*IDH1* CCA. Hepatitis B was present in one patient in the *IDH1* wild-type (3.1%) and m*IDH1* (3.2%) groups. Similarly, one patient in the *IDH1* wild-type (3.1%) and m*IDH1* (3.2%) groups had hepatitis C.

**Table 1:** Patient, disease, and testing characteristics by *IDH1* mutational status

	*IDH1* wild-type (*n* = 32)	m*IDH1* (*n* = 31)
Sex, *N*	32	31
Female, *n* (%)	18 (56.3)	16 (51.6)
Male, *n* (%)	14 (43.8)	15 (48.4)
Age at initial diagnosis (years), *N*	32	31
Mean (SD)	59.3 (10.6)	58.5 (12.0)
Median	59.9	59.6
Min, max	39.0, 80.3	29.5, 86.9
Body mass index (kg/m^2^), *N*	29	24
Median	24.0	24.9
CCA subtype at 2L, *N*	32	31
Distal extrahepatic, *n* (%)	2 (6.3)	0 (0.0)
Intrahepatic, *n* (%)	23 (71.9)	26 (83.9)
Perihilar, *n* (%)	5 (15.6)	2 (6.5)
Unknown, *n* (%)	2 (6.3)	3 (9.7)
Comorbidities, *N*	18	13
Liver cirrhosis, *n* (%)	3 (16.7)	1 (7.7)
Steatotic liver disease, *n* (%)	1 (5.6)	2 (15.4)
Diabetes, *n* (%)	5 (27.8)	4 (30.8)
Metabolic syndrome, *n* (%)	1 (5.6)	0 (0.0)
Obesity, *n* (%)	3 (16.7)	3 (23.1)
ECOG PS at 1L, *N*	29	26
0-1, *n* (%)	29 (100.0)	25 (96.2)
N/A, *n* (%)	0 (0.0)	1 (3.8)
ECOG PS at 2L, *N*	25	22
0-1, *n* (%)	24 (96.0)	20 (90.9)
2, *n* (%)	1 (4.0)	2 (9.1)
Smoking habits, *N*	32	31
Current smoker, *n* (%)	6 (18.8)	3 (9.7)
Former smoker, *n* (%)	10 (31.3)	9 (29.0)
Non-smoker, *n* (%)	14 (43.8)	13 (41.9)
Unknown, *n* (%)	2 (6.3)	6 (19.4)
Alcohol consumption, *N*	32	31
Alcohol dependent, *n* (%)	0 (0.0)	0 (0.0)
Heavy drinker, *n* (%)	3 (9.4)	0 (0.0)
Occasional drinker, *n* (%)	15 (46.9)	14 (45.2)
Abstinent, *n* (%)	7 (21.9)	7 (22.6)
Unknown, *n* (%)	7 (21.9)	10 (32.3)

*N* corresponds to the number of patients having data for the associated variable.

1L, first-line; 2L, second-line; CCA, cholangiocarcinoma; ECOG PS, Eastern Cooperative Oncology Group performance status; *IDH1*, isocitrate dehydrogenase-1; m*IDH1*, mutant *IDH1*; N/A, not applicable.

Characteristics of biomarker testing for *IDH1* mutational status are shown in Supplementary Table S1, available at https://doi.org/10.1016/j.esmogo.2026.100367. Biomarker testing was most frequently funded as part of research/clinical trials in both the *IDH1* wild-type and m*IDH1* subgroups (Supplementary Table S1, available at https://doi.org/10.1016/j.esmogo.2026.100367). Next-generation sequencing was the most frequent method for m*IDH1* testing (93.5%) and the mean (SD) time from biological sampling to result was 34.9 (63.3) days. Most patients (75.0%) with m*IDH1* had the R132C subtype (Supplementary Table S1, available at https://doi.org/10.1016/j.esmogo.2026.100367) and five (16.1%) patients with m*IDH1* had co-altered *FGFR* fusion/rearrangement.

### Treatment patterns

Before the onset of advanced/metastatic disease, a higher proportion of patients with *IDH1* wild-type CCA (53.1%) underwent surgery (predominantly involving the liver) compared with 41.9% of patients with m*IDH1* CCA. In the *IDH1* wild-type group, 25.0% of patients received radiotherapy or local therapy, whereas a notably higher percentage of patients with m*IDH1* (41.9%) were administered similar therapies.

The most frequent systemic therapy received was gemcitabine plus cisplatin at 1L (64.5% for patients with m*IDH1* CCA, 56.3% for patients with *IDH1* wild-type CCA; Table 2). Treatment interruptions during the 1L occurred in 25.8% and 37.5% of patients with m*IDH1* and *IDH1* wild-type CCA, respectively. The most frequent reason for treatment interruptions in the 1L was toxicity (45.0%).

**Table 2:** Treatment patterns by *IDH1* mutational status

	*IDH1* wild-type (*n* = 32)	m*IDH1* (*n* = 31)
First-line therapy		
Gemcitabine-cisplatin alone, *n* (%)	18 (56.3)	20 (64.5)
Gemcitabine-cisplatin plus nab-paclitaxel, *n* (%)	2 (6.3)	2 (6.5)
Capecitabine monotherapy, *n* (%)	2 (6.3)	0 (0.0)
FOLFOX, *n* (%)	2 (6.3)	0 (0.0)
Gemcitabine-cisplatin plus platinum regimen, *n* (%)	0 (0.0)	2 (6.5)
GEMOX, *n* (%)	1 (3.1)	1 (3.2)
Gemcitabine-cisplatin plus immunotherapy, *n* (%)	1 (3.1)	1 (3.2)
Gemcitabine-cisplatin plus platinum regimen plus capecitabine, *n* (%)	1 (3.1)	0 (0.0)
Gemcitabine monotherapy, *n* (%)	0 (0.0)	1 (3.2)
Gemcitabine plus capecitabine, *n* (%)	1 (3.1)	0 (0.0)
Gemcitabine plus nab-paclitaxel, *n* (%)	0 (0.0)	1 (3.2)
Gemcitabine plus platinum regimen, *n* (%)	1 (3.1)	0 (0.0)
Gemcitabine-cisplatin plus CPI-613, *n* (%)	1 (3.1)	0 (0.0)
Gemcitabine-cisplatin plus capecitabine, *n* (%)	0 (0.0)	1 (3.2)
Gemcitabine-cisplatin plus NUC-1031, *n* (%)	0 (0.0)	1 (3.2)
Gemcitabine-cisplatin plus nab-paclitaxel plus 5-FU/LV, *n* (%)	1 (3.1)	0 (0.0)
Gemcitabine-cisplatin plus platinum regimen plus NUC-1031, *n* (%)	1 (3.1)	0 (0.0)
Immunotherapy combination, *n* (%)	0 (0.0)	1 (3.2)
Second-line therapy		
FOLFOX, *n* (%)	14 (43.8)	14 (45.2)
PARP inhibitor plus immunotherapy, *n* (%)	3 (9.4)	1 (3.2)
CAPOX, *n* (%)	2 (6.3)	4 (12.9)
Capecitabine monotherapy, *n* (%)	3 (9.4)	1 (3.2)
Gemcitabine-cisplatin alone, *n* (%)	1 (3.1)	1 (3.2)
Immunotherapy monotherapy, *n* (%)	0 (0.0)	2 (6.5)
FOLFIRI plus immunotherapy, *n* (%)	2 (6.3)	0 (0.0)
FOLFIRINOX, *n* (%)	2 (6.3)	0 (0.0)
Immunotherapy combination, *n* (%)	1 (3.1)	1 (3.2)
Targeted FGFR2 therapy, *n* (%)	1 (3.1)	1 (3.2)
FOLFIRI, *n* (%)	1 (3.1)	0 (0.0)
GEMOX, *n* (%)	0 (0.0)	1 (3.2)
5-FU monotherapy, *n* (%)	0 (0.0)	1 (3.2)
5-FU plus capecitabine plus carboplatin, *n* (%)	0 (0.0)	1 (3.2)
Gemcitabine-cisplatin plus nab-paclitaxel, *n* (%)	0 (0.0)	1 (3.2)
Gemcitabine plus irinotecan, *n* (%)	0 (0.0)	1 (3.2)
Bintrafusp alfa, *n* (%)	0 (0.0)	1 (3.2)
PARP inhibitor, *n* (%)	1 (3.1)	0 (0.0)
Tazemetostat, *n* (%)	1 (3.1)	0 (0.0)

5-FU, 5-fluorouracil; CAPOX, capecitabine and oxaliplatin; CPI-613, devimistat; FGFR2, fibroblast growth factor receptor 2; FOLFIRI, folinic acid, 5-fluorouracil, and irinotecan; FOLFOX, folinic acid, 5-fluorouracil, oxaliplatin; GEMOX, gemcitabine and oxaliplatin; *IDH1*, isocitrate dehydrogenase-1; m*IDH1*, mutant *IDH1*; NUC-1031: fosgemcitabine palabenamide; PARP, poly (ADP-ribose) polymerase.

FOLFOX was the most frequently administered systemic therapy in the 2L (45.2% for patients with m*IDH1* CCA*,* 43.8% for patients with *IDH1* wild-type CCA; Table 2). During 2L, treatment interruptions occurred in 22.6% and 50.0% of patients with m*IDH1* and *IDH1* wild-type CCA, respectively. As in 1L, the most common reason for treatment interruptions in the 2L was adverse drug reactions (59.1%).

### Survival outcomes

**Figure 2:**
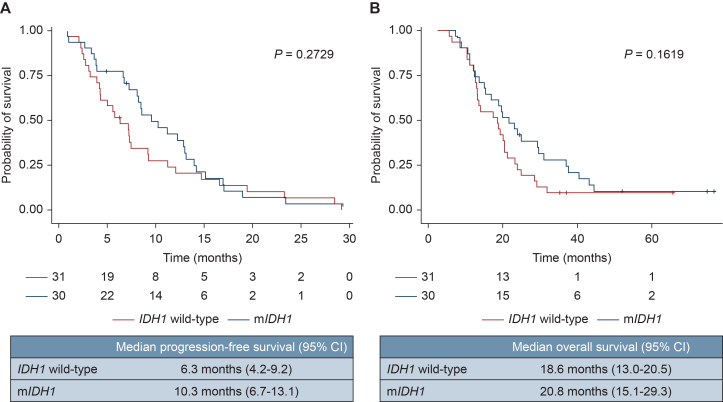
**Kaplan–Meier analysis of (A) PFS and (B) OS in patients from the start of 1L by *IDH1* mutational status.** 1L, first-line; CI, confidence interval; *IDH1*, isocitrate dehydrogenase-1; m*IDH1*, mutant *IDH1*; OS, overall survival; PFS, progression-free survival.

**Figure 3:**
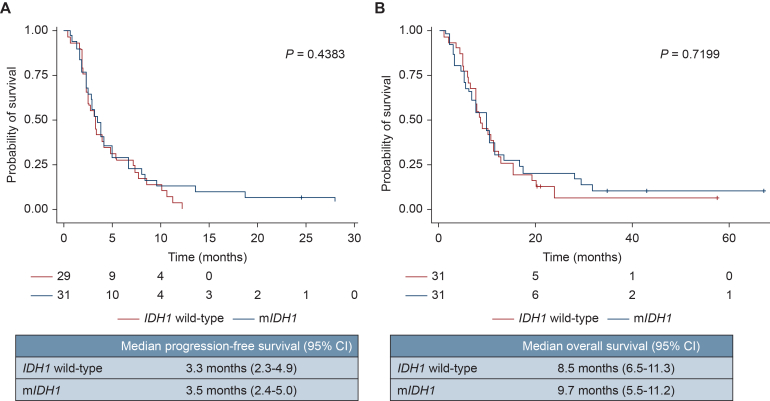
**Kaplan–Meier analysis of (A) PFS and (B) OS in patients from the start of 2L by *IDH1* mutational status.** 2L, second-line; CI, confidence interval; *IDH1*, isocitrate dehydrogenase-1; m*IDH1*, mutant *IDH1*; OS, overall survival; PFS, progression-free survival.

The median PFS from the start of 1L was 10.3 (95% CI 6.7-13.1) months for the m*IDH1* group and 6.3 (95% CI 4.2-9.2) months for the *IDH1* wild-type group (Figure 2A). The median OS from the start of 1L was 20.8 (95% CI 15.1-29.3) months for the m*IDH1* group and 18.6 (95% CI 13.0-20.5) months for the *IDH1* wild-type group (Figure 2B). The median PFS from the start of 2L was 3.5 (95% CI 2.4-5.0) and 3.3 (95% CI 2.3-4.9) months for the m*IDH1* and *IDH1* wild-type groups, respectively (Figure 3A). The median OS from the start of 2L was 9.7 (95% CI 5.5-11.2) months for patients with m*IDH1* CCA and 8.5 (95% CI 6.5-11.3) months for patients with *IDH1* wild-type CCA (Figure 3B).

Univariate analysis showed a statistically significant adverse impact of FOLFOX during 2L (HR 1.78, 95% CI 1.04-3.04, *P* = 0.034) and a positive impact of therapy with a gemcitabine-platinum compound during 1L (HR 0.48, 95% CI 0.24-0.98, *P* = 0.043) on median OS. Both observations remained statistically significant in the multivariate Cox proportional hazards model. The univariate analysis showed no statistically significant impact of *IDH1* mutational status on median OS (HR 0.72, 95% CI 0.42-1.22, *P* = 0.220), and this remained non-significant when adjusting for multiple factors in the multivariate analysis. Univariate analysis showed no statistically significant impact of any variable (including *IDH1* mutational status) on median PFS at 1L. In the univariate analysis of median PFS at 2L, FOLFOX during 2L had a negative impact (HR 1.92, 95% CI 1.13-3.28, *P* = 0.016), and surgery had a positive impact (HR 0.57, 95% CI 0.33-0.99, *P* = 0.045). Only FOLFOX during 2L remained statistically significant in the multivariate analysis. *IDH1* mutational status had no impact on median PFS at 2L (HR 0.81, 95% CI 0.48-1.38, *P* = 0.442).

For patients receiving a gemcitabine-platinum regimen at 1L (26 m*IDH1*; 26 *IDH1* wild-type), the median PFS from the start of 1L was 11.2 months (95% CI 6.7-13.1) in the m*IDH1* group and 7.2 months (95% CI 4.3-11.3) in the *IDH1* wild-type group (Supplementary Figure S2A, available at https://doi.org/10.1016/j.esmogo.2026.100367). The median OS from the start of 1L was 23.6 months (95% CI 13.7-31.0) and 19.0 months (95% CI 13.0-21.3), respectively (Supplementary Figure S2B, available at https://doi.org/10.1016/j.esmogo.2026.100367). For patients receiving FOLFOX at 2L (14 m*IDH1*; 14 *IDH1* wild-type), the median PFS from the start of 2L was 2.9 (95% CI 1.4-3.9) and 2.7 (95% CI 1.9-7.2) months in the m*IDH1* and *IDH1* groups, respectively (Supplementary Figure S3A, available at https://doi.org/10.1016/j.esmogo.2026.100367). Median OS from the start of 2L for patients receiving FOLFOX at 2L was 8.0 (95% CI 3.0-10.5) months for the m*IDH1* group and 6.3 (95% CI 3.4-8.9) months for the *IDH1* wild-type group (Supplementary Figure S3B, available at https://doi.org/10.1016/j.esmogo.2026.100367).

Partial response per RECIST version 1.1^18^ was achieved in 5 (21.7%) of the 23 patients with m*IDH1* CCA with evaluable imaging in 1L and in 5 (31.3%) of the 16 patients with *IDH1* wild-type CCA with evaluable imaging in the 1L. One (10.0%) of the 10 patients with m*IDH1* and 2 (15.4%) of the 13 patients with *IDH1* wild-type CCA with evaluable imaging during 2L achieved a partial response per RECIST version 1.1 (Supplementary Figure S4, available at https://doi.org/10.1016/j.esmogo.2026.100367).^18^ No patients in either subgroup in 1L or 2L achieved a complete response.

### Medical resource utilization

Overall, medical resource utilization was similar between patient groups (Supplementary Table S2, available at https://doi.org/10.1016/j.esmogo.2026.100367). The most frequent type of ward for inpatient admission was internal medicine/general ward (57.1% and 25.0% in m*IDH1* and *IDH1* wild-type groups, respectively). The most frequent ward for outpatient care was an emergency ward (40.0%) in the m*IDH1* group, while only one patient received outpatient care in the *IDH1* wild-type group, which was an oncology consultation.

### Safety outcomes

During 1L therapy, 17 patients in the m*IDH1* group and 17 patients in the *IDH1* wild-type group had at least one reported adverse drug reaction. During 2L, 17 patients in the m*IDH1* group and 21 patients in the *IDH1* wild-type group had at least one reported adverse drug reaction (Supplementary Table S3, available at https://doi.org/10.1016/j.esmogo.2026.100367). The most frequent adverse reactions reported in both groups irrespective of line of therapy (excluding ‘other’) were anemia (5.9% in patients with m*IDH1* CCA and 15.2% in patients with *IDH1* wild-type CCA) and fatigue (9.7% and 12.0%, respectively), which were consistent with the label of the administered regimens. Most reported adverse drug reactions were grade 1 at both 1L and 2L (61.0% in patients with m*IDH1* CCA and 66.8% in patients with *IDH1* wild-type CCA.

## Discussion

This international, multicenter, retrospective, observational study provides meaningful real-world data on the natural disease course of patients with CCA treated with standard-of-care therapies before the availability of ivosidenib, focusing on m*IDH1* and *IDH1* wild-type groups. Real-world baseline characteristics and medical resource utilization were similar between patients with m*IDH1* and *IDH1* wild-type CCA.

The impact of m*IDH1* on prognosis in CCA has been debated. Some studies have shown that patients with m*IDH1* CCA have a poorer prognosis than patients with *IDH1* wild-type disease.^19^^,^^20^ However, most studies have found no prognostic impact of m*IDH1* in CCA,^21^, ^22^, ^23^, ^24^, ^25^, ^26^ which is consistent with the results of this study, but the small sample size may limit the accuracy of this assessment. While these previous studies only documented the lack of prognostic significance of m*IDH1* in CCA, this study also assessed the outcomes of patients with m*IDH1* and *IDH1* wild-type CCA treated with standard 1L and 2L therapies. In this descriptive analysis, patients with m*IDH1* disease had longer PFS and OS than patients with *IDH1* wild-type disease, despite fewer patients in the m*IDH1* group achieving a partial response than the *IDH1* wild-type group. More patients in the *IDH1* wild-type group (16.7%) had cirrhosis than patients in the m*IDH1* group (7.7%), which may have had an impact on survival outcomes. Cirrhosis has previously been found to confer a poorer prognosis in patients with CCA,^27^ but a recent study reported that cirrhosis is not an independent factor for survival in patients with iCCA.^28^ However, these results should be interpreted with caution due to the small sample size and proportion of patients with cirrhosis, bias in the selection of patients fit enough to be considered for 2L treatment, and most patients receiving treatment in the context of clinical trials. The OS and PFS reported in this study were longer than expected, potentially due to this selection of patients fit enough to commence 2L treatment and the high proportion of patients undergoing surgery. Further research is needed to assess the impact of m*IDH1* on survival outcomes in response to standard-of-care therapies in a larger, unbiased sample of patients with CCA in the real world. The phase 3b, real-world, prospective ProvIDHe study of patients with m*IDH1* CCA receiving ivosidenib is currently ongoing,^29^, ^30^, ^31^ and could demonstrate the impact of ivosidenib on outcomes in the m*IDH1* CCA population.

In the EvIDHence study, biomarker testing in patients with CCA was mainly carried out in a research/clinical trial setting rather than as standard medical practice, which is consistent with the patient management at the time of the study (before December 2021). The limited targeted treatment options for patients with CCA at the time of this study may explain the lack of standardized routine testing and variability in timing of testing and results. With the growing number of approved targeted therapies for CCA, biomarker testing is now included as standard-of-care according to international guidelines and is used to help guide treatment strategies,^4^^,^^5^^,^^32^ which may have led to improvements in waiting times for biomarker testing and results. Other molecular alterations, such as *FGFR2* fusion/rearrangement, were similar between patients with m*IDH1* and *IDH1* wild-type CCA. Previous studies have shown that m*IDH1* co-occurs with *FGFR2* in a minority of patients with iCCA.^33^^,^^34^

The main limitation of this study was that it had a small sample size. Patient enrolment was restricted because most of the identified patients with m*IDH1* CCA had already been treated with an mIDH1 inhibitor in the context of a managed program or in clinical trials. Moreover, the patients selected for inclusion in this study had survived 1L and were fit enough to initiate 2L, which is demonstrated by the young median age of the cohort and most patients having an ECOG PS of 0-1 at start of 1L and 2L. Therefore, this was a source of immortal time bias that may have resulted in an overestimation of survival outcomes. The limited patient numbers in this study did not allow for the establishment of *IDH1* mutations as prognostic biomarkers. Further studies, such as ProvIDHe,^31^ are required to assess the prognostic role of m*IDH1* on outcomes in patients with CCA. We recognize that other molecular alterations may also have impacted prognosis, but the small sample size meant that the study was not powered to examine these factors. The study eligibility period preceded the introduction of immune checkpoint inhibitors in 1L; therefore, the applicability of the study results to current clinical practice may be limited. Despite this, the findings provide a valuable contribution to the understanding of patterns and outcomes in patients with m*IDH1* and *IDH1* wild-type CCA in the pretargeted therapy era. As is the case with most studies relying on existing medical records, some information may not be available for all patients, especially for deceased patients whose archived medical records may not be fully accessible.

### Conclusions

This retrospective, real-world study examined the treatment sequences and outcomes in patients with CCA before the arrival of targeted therapies and highlighted no difference in the efficacy of 1L and 2L therapies or OS in m*IDH1* and *IDH1* wild-type CCA. Despite its limitations, the EvIDHence study provides a valuable real-world snapshot of treatment patterns and outcomes of patients with m*IDH1* and *IDH1* wild-type CCA before the arrival of mIDH1 inhibitors. Prospective, real-world studies are ongoing to further assess the impact of mIDH1 inhibitors, such as ivosidenib, in patients with m*IDH1* CCA.
