# Supplementary material for: The EvIDHence study: real-world treatment patterns and safety outcomes in mutant and wild-type IDH1 cholangiocarcinoma before availability of mIDH1 inhibitors
Source: ESMO Gastrointest Oncol. 2026 Jul 23;13(Pt B):100367. doi: 10.1016/j.esmogo.2026.100367 (PMC13459570; doi:10.1016/j.esmogo.2026.100367)
Supplement: Supplementary file 1 [file mmc1.docx]

**Supplementary Materials
Supplementary Table 1.** Tumor and testing characteristics by *IDH1* mutational status

|  | ***IDH1* wild-type (n = 32)** | **m*IDH1*  (n = 31)** |
| --- | --- | --- |
| *IDH1* allele subtypes - N | 32 | 31 |
| R132C, n (%) | - | 24 (75.0) |
| R132G, n (%) | - | 1 (3.1) |
| Unknown | - | 5 (15.6) |
| Molecular alterations - N* | 37 | 37 |
| *BRCA* 1/2 mutation, n (%) | 0 (0.0) | 1 (2.7) |
| *ERBB2* mutation*,* n (%) | 1 (2.7) | 0 (0.0) |
| *FGFR2* fusion/rearrangement, n (%) | 5 (13.5) | 4 (10.8) |
| *NTRK* fusion*,* n (%) | 1 (2.7) | 0 (0.0) |
| *PIK3CA* mutation*,* n (%)  Other, n (%)  Unknown, n (%) | 0 (0.0)  30 (81.1)  0 (0.0) | 5 (13.5)  20 (54.1)  7 (18.9) |
| Methodology used to detect *IDH1* mutation - N | 32 | 30 |
| NGS, n (%) | 31 (96.9) | 27 (90.0) |
| Immunohistochemistry, n (%) | 1 (3.1) | 0 (0.0) |
| Other, n (%) | 0 (0.0) | 3 (10.0) |
| Funding source for mutation testing - N | 32 | 31 |
| Insurance, n (%) | 7 (21.9) | 7 (22.6) |
| Public, n (%) | 6 (18.8) | 4 (12.9) |
| Research project or trial, n (%) | 19 (59.4) | 20 (64.5) |
| Time from biological sample collection to the results of the IDH1 testing (days) - N | 32 | 30 |
| Mean (SD) | 32.8 (63.6) | 37.1 (64.0) |
| Median (min, max) | 17.0 (1.0, 365.0) | 21.5 (1.0, 365.0) |

N corresponds to the number of patients having data for the associated variable unless stated otherwise. *Number of events. *IDH1*, isocitrate dehydrogenase-1. m*IDH1*, mutant *IDH1*. *BRCA*, Breast Cancer gene. *ERBB2*, erythroblastic oncogene B 2. *FGFR2*, Fibroblast growth factor receptor 2. Max, maximum. Min, minimum. NGS, next-generation sequencing; *NTRK*, Neurotrophic tyrosine receptor kinase. *PIK3A*, Phosphatidylinositol-4,5-bisphosphate 3-kinase, catalytic subunit alpha.

**Supplementary Table 2.** Medical resource utilization during 2L by *IDH1* mutational status

|  | ***IDH1* wild-type  (n = 32)** | **m*IDH1***  **(n = 31)** |
| --- | --- | --- |
| Total number of routine visits to the site during 2L - N | 26 | 25 |
| Mean (SD) | 9.8 (10.2) | 10.4 (10.6) |
| Patients undergoing inpatient admission during 2L - N | 32 | 31 |
| Yes, n (%) | 7 (21.9) | 7 (22.6) |
| No, n (%) | 20 (62.5) | 19 (61.3) |
| Unknown, n (%) | 5 (15.6) | 5 (16.1) |
| Reason for inpatient admission - N | 7 | 7 |
| Administration of systemic therapy regimen, n (%) | 0 (0.0) | 1 (14.3) |
| General disease management, n (%) | 4 (57.1) | 3 (42.9) |
| Management of a grade 3/4 adverse drug reaction, n (%) | 0 (0.0) | 1 (14.3) |
| Other, n (%) | 3 (42.9) | 2 (28.6) |
| Patients undergoing outpatient care during 2L - N | 32 | 31 |
| Yes, n (%) | 1 (3.1) | 3 (9.7) |
| No, n (%) | 25 (78.1) | 23 (74.2) |
| Unknown, n (%) | 6 (18.8) | 5 (16.1) |
| Patients having office visits during 2L - N | 32 | 31 |
| Yes, n (%) | 10 (31.3) | 12 (38.7) |
| No, n (%) | 17 (53.1) | 13 (41.9) |
| Unknown, n (%) | 5 (15.6) | 6 (19.4) |
| Type of specialist* – N | 10 | 12 |
| Gastroenterologist, n (%) | 2 (7.6) | 1 (2.4) |
| Medical oncologist, n (%) | 6 (23.1) | 29 (69.0) |
| General practitioner, n (%) | 1 (3.8) | 3 (7.1) |
| Other, n (%) | 17 (65.4) | 9 (21.4) |
| Patient undergoing any imaging exams during 2L – N | 32 | 31 |
| Yes, n (%) | 26 (81.3) | 28 (90.3) |
| No, n (%) | 6 (18.8) | 2 (6.5) |
| Types of imaging exams performed during 2L - N | 26 | 28 |
| Computed tomography scan, n (%) | 25 (67.6) | 28 (71.8) |
| Magnetic resonance imaging, n (%) | 4 (10.8) | 3 (7.7) |
| Positron emission tomography scan, n (%) | 0 (0.0) | 3 (7.7) |
| Ultrasound, n (%) | 4 (10.8) | 2 (5.1) |
| X-ray, n (%) | 3 (8.1) | 3 (7.7) |
| Other, n (%) | 1 (2.7) | 0 (0.0) |

*N corresponds to the number of patients that had data available and counts each visit to a specialist individually for each patient. 2L, second-line. *IDH1*, isocitrate dehydrogenase-1. m*IDH1*, mutant *IDH1*.

**Supplementary Table 3.** Adverse drug reactions

|  | ***IDH1* wild-type**  **(n = 32)** | **m*IDH1***  **(n = 31)** |
| --- | --- | --- |
| Number of patients with at least one adverse drug reaction at first-line | 17 | 17 |
| Adverse drug reactions at first-line | | |
| Anemia, n (%) | 16 (17.2) | 9 (7.8) |
| Fatigue, n (%) | 13 (14.0) | 11 (9.5) |
| Nausea, n (%) | 8 (8.6) | 8 (6.9) |
| Decreased appetite, n (%) | 4 (4.3) | 2 (1.7) |
| Diarrhea, n (%) | 3 (3.2) | 3 (2.6) |
| Vomiting, n (%) | 2 (2.2) | 3 (2.6) |
| Alopecia, n (%) | 2 (2.2) | 1 (0.9) |
| Edema, n (%) | 1 (1.1) | 3 (2.6) |
| Rash, n (%) | 1 (1.1) | 2 (1.7) |
| Abdominal pain, n (%) | 1 (1.1) | 2 (1.7) |
| Pyrexia, n (%) | 0 (0.0) | 2 (1.7) |
| Dyspnea, n (%) | 1 (1.1) | 0 (0.0) |
| Other, n (%) | 41 (44.1) | 70 (60.3) |
| Adverse drug reaction severity at first-line |  |  |
| Grade 1, n (%) | 65 (69.9) | 66 (56.9) |
| Grade 2, n (%) | 22 (23.7) | 36 (31.0) |
| Grade 3, n (%) | 5 (5.4) | 12 (10.3) |
| Grade 4, n (%) | 1 (1.1) | 2 (1.7) |
| Grade 5, n (%) | 0 (0.0) | 0 (0.0) |
| Number of patients with at least one adverse drug reaction at second-line | 21 | 17 |
| Adverse drug reactions at second-line | | |
| Fatigue, n (%) | 13 (10.5) | 12 (10.0) |
| Anemia, n (%) | 17 (13.7) | 5 (4.2) |
| Nausea, n (%) | 9 (7.3) | 5 (4.2) |
| Diarrhea, n (%) | 3 (2.4) | 6 (5.0) |
| Decreased appetite, n (%) | 4 (3.2) | 3 (2.5) |
| Vomiting, n (%) | 4 (3.2) | 1 (0.8) |
| Rash, n (%) | 2 (1.6) | 2 (1.7) |
| Alopecia, n (%) | 3 (2.4) | 0 (0.0) |
| Edema, n (%) | 1 (0.8) | 2 (1.7) |
| Abdominal pain, n (%) | 0 (0.0) | 2 (1.7) |
| Ascites, n (%) | 0 (0.0) | 2 (1.7) |
| Febrile neutropenia, n (%) | 0 (0.0) | 1 (0.8) |
| Leukocytosis, n (%) | 1 (0.8) | 0 (0.0) |
| Dyspnea, n (%) | 1 (0.8) | 0 (0.0) |
| Pyrexia, n (%) | 1 (0.8) | 0 (0.0) |
| Other, n (%) | 65 (52.4) | 79 (65.8) |
| Adverse drug reaction severity at second-line |  |  |
| Grade 1, n (%) | 80 (64.5) | 78 (65.0) |
| Grade 2, n (%) | 43 (34.7) | 34 (28.3) |
| Grade 3, n (%) | 1 (0.8) | 7 (5.8) |
| Grade 4, n (%) | 0 (0.0) | 1 (0.8) |
| Grade 5, n (%) | 0 (0.0) | 0 (0.0) |

*IDH1*, isocitrate dehydrogenase-1. m*IDH1*, mutant *IDH1*.

**Supplementary Figure 1.** Flowchart of patients screened for inclusion in this study

Screened population (n = 74)

Inclusion criteria not met (n = 4)

- Advanced or metastatic CCA, irrespective of its subtype: iCCA, pCCA, dCCA, or unknown subtype (n = 1)
- Initiation of a 2L systemic therapy during the study eligibility period (n = 3)

Patients meeting none of the exclusion criteria (n = 64)

Patients excluded by protocol deviation (n = 1)

- 1L treatment only received for one day (n = 1)

Patients included in study (n = 63)

Patients meeting all inclusion criteria (n = 70)

Exclusion criteria met (n = 6)

- Patients who received any investigational (USA only: investigational or marketed) IDH1 inhibitor at any line of CCA treatment (n = 6)

**Supplementary Figure 2.** Kaplan-Meier analysis of (**A**) progression-free survival and (**B**) overall survival in patients receiving 1L gemcitabine-platinum regimen from start of 1L by *IDH1* mutational status

**A**

*P*=0.5245

|  | **Median progression-free survival (95% CI)** |
| --- | --- |
| *IDH1* wild-type | 7.2 months (4.3-11.3) |
| m*IDH1* | 11.2 months (6.7-13.1) |

**B**

*P*=0.1966

|  | **Median overall survival (95% CI)** |
| --- | --- |
| *IDH1* wild-type | 19.0 months (13.0-21.3) |
| m*IDH1* | 23.6 months (13.7-31.0) |

1L, first-line. *IDH1*, isocitrate dehydrogenase-1. m*IDH1*, mutant *IDH1*.

**Supplementary Figure 3.** Kaplan-Meier analysis of (**A**) progression-free survival and (**B**) overall survival in patients receiving 2L FOLFOX from start of 2L by *IDH1* mutational status

**A**

*P*=0.3295

|  | **Median progression-free survival (95% CI)** |
| --- | --- |
| *IDH1* wild-type | 2.7 months (1.9-7.2) |
| m*IDH1* | 2.9 months (1.4-3.9) |

**B**

*P*=0.1471

|  | **Median overall survival (95% CI)** |
| --- | --- |
| *IDH1* wild-type | 6.3 months (3.4-8.9) |
| m*IDH1* | 8.0 months (3.0-10.5) |

2L, second-line. FOLFOX, folinic acid, 5-fluorouracil, oxaliplatin*. IDH1*, isocitrate dehydrogenase-1. m*IDH1*, mutant *IDH1*.

**Supplementary Figure 4.** Patients with evaluable imaging that achieved partial response and stable disease in 1L and 2L according to treatment group

Percentage of patients (%)

|  | IDH1 wild-type | mIDH1 |
| --- | --- | --- |
| Partial response (1L) n = 16 | 31.3 | 21.7 |
| Stable disease (1L) n = 23 | 68.8 | 78.3 |
| Partial response (2L) n = 13 | 15.4 | 10 |
| Stable disease (2L) n = 10 | 84.6 | 90 |

The data presented here are from patients with evaluable imaging and response was evaluated according to RECIST version 1.1^18^. 1L, first-line. 2L, second-line. *IDH1*, isocitrate dehydrogenase-1. m*IDH1*, mutant *IDH1*.
